# The Evolutionary History and Spatiotemporal Dynamics of the NC Lineage of Citrus Tristeza Virus

**DOI:** 10.3390/v9100272

**Published:** 2017-10-12

**Authors:** María José Benítez-Galeano, Matías Castells, Rodney Colina

**Affiliations:** Laboratorio de Virología Molecular, Centro Universitario Regional Litoral Norte (CENUR Litoral Norte), Universidad de la República (UdelaR), Rivera 1350, 50000 Salto, Uruguay; mbenitezgaleano@gmail.com (M.J.B-G.); matiascastellsbauer@gmail.com (M.C.)

**Keywords:** citrus tristeza virus, evolution rate, phylogeography, NC genotype

## Abstract

Citrus tristeza virus (CTV) is a major pathogen affecting citrus trees worldwide. However, few studies have focused on CTV’s evolutionary history and geographic behavior. CTV is locally dispersed by an aphid vector and long distance dispersion due to transportation of contaminated material. With the aim to delve deeper into the CTV-NC (New Clade) genotype evolution, we estimated an evolution rate of 1.19 × 10^−3^ subs/site/year and the most common recent ancestor in 1977. Furthermore, the place of origin of the genotype was in the United States, and a great expansion of the population was observed in Uruguay. This expansion phase could be a consequence of the increment in the number of naïve citrus trees in Uruguayan orchards encompassing citrus industry growth in the past years.

## 1. Introduction

RNA viruses have a great potential for rapid evolution due to the high mutation rates, large population sizes, and short generation times [1]. This rapid evolution means that epidemiological and evolutionary processes occur on a time scale of a few years.

Citrus tristeza virus (CTV; genus *Closterovirus*, family *Closteroviridae*) is one of the most destructive pathogens that affect citrus trees worldwide [2]. The CTV genome is a single-stranded positive sense RNA molecule of approximately 19.3 kb in length containing 12 open reading frames that encodes for at least 19 protein products, including replication proteins, cell-to-cell movement proteins, virion assembly proteins, suppressors of RNA silencing proteins, and two capsid proteins [3,4,5,6]. p25 is a multifunctional protein involved in the suppression of intercellular RNA silencing and works as a capsid protein covering 95% of the virus genome [3,6].

Natural CTV hosts essentially include species of the genera *Citrus* and *Fortunella,* and depending on the virus strains and on the species or scion–rootstock combinations, CTV may cause three distinct syndromes named quick decline, stem pitting, and seedling yellows [2]. As an aphid-borne virus transmitted in a semi-persistent manner, CTV is mainly transmitted by vector species from the genera *Toxoptera* and *Aphis*, which locally spread the virus within a given citrus producing area. Long-distance dissemination is caused principally by the transportation of infected material [2].

Like most RNA viruses, CTV exists in nature as a mixture of genetic variants that coexist in the same plant displaying high levels of genetic and phenotypic diversity [7]. Genetic studies of different strains of CTV revealed the existence of seven distinct genetic lineages or genotypes worldwide, known as VT, T3, T30, T36, T68, RB, and NC (New Clade) the latter being proposed by our group [8,9,10,11,12,13]. This new genotype includes the HA16-5 (GQ454870) and Taiwan-Pum/M/T5 (JX266713) strains, from which the complete genomes are available, grouping into a clade with nucleotide sequence identities higher than 98.7% for *p25* gene between strains [13].

CTV biology has been intensively studied for many decades, but evolutionary and spatiotemporal phylodynamic studies, which can provide relevant information for understanding the emergence of new viral diseases, genotypes, or genetic variants and for designing more efficient strategies for disease control, have not been conducted in-depth. In this matter, few studies have focused on CTV’s evolutionary history with reference to geography [12,14,15]. The whole CTV genome evolutionary rate, from an infectious clone introduced by bark-flap inoculation into *C. macrophylla*, has been calculated to be 7 years after inoculation and was in the order of 10^−5^ substitutions per site per year (s/s/y) [12]. This parameter was also calculated for *p20* and *p25* genes, in both cases resulting one order of magnitude higher (10^−4^ s/s/y) than that for the complete genome estimation [14,15]. In the same article, Silva and co-workers [14] estimated the time of the most recent common ancestor (tMRCA) of CTV lineages based on the *p25* gene. Nevertheless, these parameters were estimated for all CTV extant genotypes described at the moment of publication of the respective articles, highlighting the fact that the NC genotype has not yet been described. Another important contribution regarding the geographic behavior of CTV was made by Davino and co-workers [15], who studied the genetic relationship among CTV strains from Sicily, Italy, and evaluated the geographical and temporal dissemination of the virus in the island. Their results suggest that mild and severe CTV isolates belonging to five different clades (lineages) were introduced in Sicily in 2002.

With the aim to describe the spatiotemporal dynamics of the new NC-CTV genotype, which is highly represented in Uruguayan citrus orchards, we performed a Bayesian analysis of 123 dated sequences belonging to the aforementioned genotype. Sequences from Africa, Asia, Europe, North America, and South America were included in the analysis, and the ancestor of the NC genotype was dated as well as its evolutionary rate based on the p25 coding region. We also delineated the possible geographic dispersion route of the virus since its origin until 2015. To our knowledge, this is the first exhaustive report of the evolutionary and geographical behavior of the NC genotype, from which an exhaustive characterization remains incomplete.

## 2. Materials and Methods

### 2.1. Sequence Dataset

A total of 123 sequences of CTV *p25* gene and associated information including collection date and location were retrieved from the NCBI nucleotide database (http://www.ncbi.nlm.nih.gov) to perform the analyses. The dataset comprises sequences collected between 1979 and 2015 from different countries, including Angola, Argentina, Brazil, China, Greece, India, Portugal, Sao Tomé and Principe, Taiwan, United States, and Uruguay. The Uruguayan sequences used in the present work were obtained from samples processed by our group (Table S1).

### 2.2. Phylogenetic Analysis

CTV sequences were aligned with ClustalW in MEGA 6.0 (available online: http://www.megasoftware.net/) generating a sequence alignment of 572 nucleotides in length covering positions 16130–16702 (NCBI nucleotide sequence accession number U56902) [16].

In order to prevent bias in the phylogenetic, phylodynamic, and phylogeographic analyses, we used the RDP, GENECONV, MAXCHI, CHIMAERA, 3SEQ, BOOTSCAN, LARD, and SISCAN heuristic recombination detection methods implemented in the RDP4 software package with default settings [17,18,19,20,21,22,23,24,25]. No recombinant sequences were identified, nor was there any evidence for phylogenetic incongruence among the sequences analyzed here.

The model of nucleotide substitution that best fit the dataset (HKY) was selected using the jModelTest program according to the Akaike Information Criterion (AIC) [26,27]. Maximum Likelihood (ML) phylogenetic trees were reconstructed with PhyML program using an online web server [28,29]. The heuristic tree search was performed using the SPR branch-swapping algorithm and branch support was calculated with the approximate likelihood-ratio (aLRT) SH-like test [30].

### 2.3. Phylodynamic and Phylogeographic Approaches

The time of most recent common ancestor (tMRCA), the evolutionary rate of the new CTV genotype (NC) and its demographic history in Uruguay were jointly inferred using the Bayesian Markov Chain Monte Carlo (MCMC) statistical framework implemented in the BEAUti/BEAST package v1.8.0 (available online: http://tree.bio.ed.ac.uk/software/beast/) [31].

The TempEst program was used to determine that sequences used in this work showed temporal structure to proceed with molecular clock analyses.

The HKY nucleotide substitution model was determined to be the best fit with the dataset and the lognormal relaxed (uncorrelated) molecular clock model was used [14,32,33]. Bayesian analyses were conducted with the available tree priors described by Silva and co-workers [14] for the *p25* gene and the best demographic model was evaluated through Bayes Factor; the Bayesian Skyline coalescent tree prior was used [34]. A Markov Chain Monte Carlo (MCMC) process was run for 200 million generations, and the results were visualized with Tracer v1.5.0 program (available from http://beast.bio.ed.ac.uk/Tracer) discarding the initial 10% of the run as burn-in. The effective sample size (ESS) values were checked to evaluate the convergence of the analysis, accepting only values higher than 200 for all the parameters. The demographic history of the new CTV clade in Uruguay was represented graphically with Tracer v1.5.0, through the effective number of infections as a function of time.

Phylogeographic analysis was conducted with HKY nucleotide substitution model, lognormal relaxed (uncorrelated) molecular clock model and a Bayesian skyline coalescent tree prior. Using the evolutionary rate estimated in this work and a Bayesian stochastic search variable selection (BSSVS), MCMC processes were run for 200 million generations. ESS values were checked to evaluate the convergence of the analysis, accepting only values higher than 200 for all the parameters. The Maximum Credibility Clade Tree (MCCT) was obtained with TreeAnnotator v1.8.0 of BEAUti/BEAST package and visualized with FigTree v 1.4.0 (http://tree.bio.ed.ac.uk/software/figtree). Phylogeographic dissemination pattern was visually determined with SPREAD v 1.0.6 and KMZ file was visualized with Google Earth^®^ [35]. The KMZ file is available from the authors upon request.

## 3. Results

### 3.1. The Monophyly and Temporal Signal of the Data Set

In order to test that the Uruguayan field samples collected in 2015 belonging to the NC genotype, a phylogenetic analysis with the ML method was performed, and the tree showed that all the sequences used in this work clustered as a highly supported monophyletic group (aLRT = 1) (Figure S1). Another important fact to take into account when Bayesian approaches are used is that the dataset must have temporal signal and are not the result of artefactual nucleotide changes in the sequences [36,37]. We estimated the temporal signal with TempEst software (available online: http://tree.bio.ed.ac.uk/software/tempest/) [38] and sequences with incongruent data/variability were discarded.

### 3.2. Rates and Dates of Evolution

**Figure 1:**
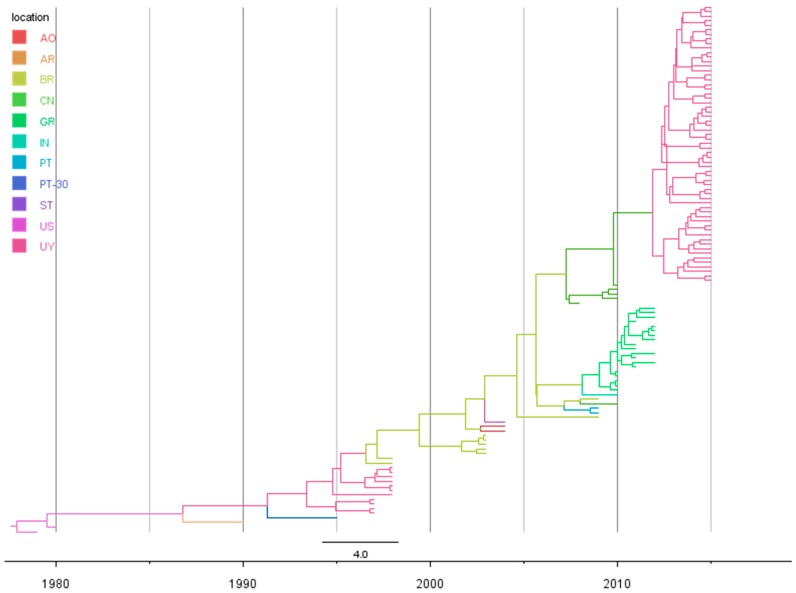
Time-scaled Bayesian MCC phylogeny of CTV-NC genotype. Sequences from different countries encompassing the *p25* gene were used to phylogeny reconstruction. Branches were colored according to the most probable location of their parental nodes. AO: Angola; AR: Argentina; BR: Brazil; CN: China; GR: Greece; IN: India; PT: Portugal; PT-30: Madeira Island; ST: Sao Tomé and Príncipe; US: United States; UY: Uruguay.

Our Bayesian coalescent estimates of evolutionary dynamics, based on the *p25* gene, indicate that the CTV-NC genotype evolves at a rate of 1.19 × 10^−3^ subs/site/year (95% HPD, 1.72 × 10^−3^ − 7.1 × 10^−4^ subs/site/year). This rate was used to estimate times to common ancestry. Based on the MCCT (Figure 1), the most plausible place of origin of the new CTV-NC genotype data was the United States (US), 37 years from the most recent analyzed strain (2015), dating back to 1977 with a narrow 95% Highest Posterior Density (HPD) interval ranging from 1974 to 1979.

**Figure 2:**
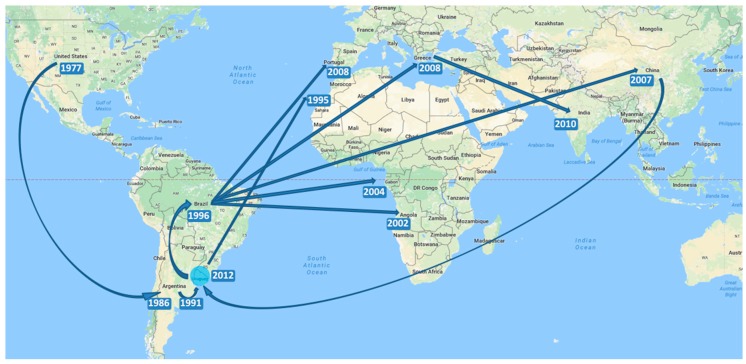
Spatiotemporal dynamics of CTV-NC genotype circulating in the world. Dispersal pattern (blue arrows) between 1977 and 2015, reconstructed from the MCC tree and the probably year of arrival to each country (blue squares with the year) are shown. The local expansion occurred in Uruguay is shown (light blue circle). Map was adapted from Google Maps^®^ (Google®, Mountain View, California, United States).

With respect to the spatio-temporal dynamics of the genotype, the results are summarized on an MCC tree with branches colored according to the most probable location of their parental nodes (Figure 1), and with a map representing the most significant migration routes among countries between 1977 and 2015 (Figure 2).

As can be seen in both Figure 1 and Figure 2, after its origin in 1977, the virus began to move from the US to different countries. In 1986 (1980–1990) NC-genotype viruses spread to Argentina and 5 years later, in 1991 (1987–1994) the virus arrived to Uruguay from its neighboring country (Figure 2). After 1991, the virus moved from Uruguay to Madeira Island in Portugal and to Brazil in 1996 (1994–1998) (Figure 2). After its arrival in 1996 to Brazil, the virus continued spreading to Angola in 2002 (2001–2004) and to Sao Tomé and Príncipe (Figure 2). In 2007, the virus genotype moved from Brazil to Asia, specifically to China with a 95% HPD interval ranging from 2006 to 2008. In 2008, the virus moved from Brazil to mainland Portugal (2007–2009) and to Greece (2005–2009), and from the latter moved to India after 2008 (Figure 2). Finally, a second introduction of the virus from China to Uruguay occurred in 2012 (2010–2013) with an important local expansion of the population after its arrival (Figure 2).

### 3.3. Evolutionary History of the Genotype

**Figure 3:**
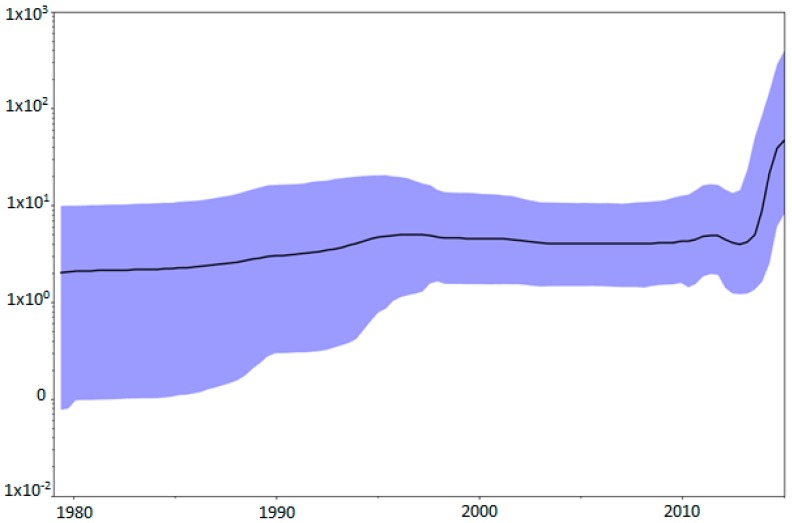
Population dynamics of the CTV-NC genotype. The demographic history of the genotype is shown since its origin in 1977 until the most recent sequence of the dataset, 2015. The Bayesian Skyline plot shows the evolution in population size. Median (dark line) and upper and lower 95% HPD (blue region) estimates of effective population size (*y*-axis) through time in years (*x*-axis) are shown.

The demographic reconstruction of this new genotype, observed in the Skyline plot (Figure 3), showed that the viral population size can be divided into two different phases.

The first phase shows that the population size remained constant since its origin until around 2012. In the second phase, we can observe an increment of one log of the population size around this date until 2015, the date of the most recent sequences of our dataset, suggesting that the population is growing.

## 4. Discussion

The monophyly of the NC genotype jointly with the fact that no recombinant sequences were found among the strains used in the present work are important in order to avoid bias in the subsequent phylodynamic and phylogeographic analyses.

The substitution rate obtained in the present work is similar to values reported for other RNA viruses [39]. Nevertheless, Silva and co-workers [14] recently described the gene evolutionary rate in the order of 10^−4^ subs/site/year, although their calculations were made for all the CTV circulating genotypes at once. It has been proposed that, with the emergence of a new variant or during an outbreak, the evolutionary rate of a virus is faster and becomes slower and reaches the equilibrium after a period of time [40,41]. In this case, this is the first calculation of the CTV-NC genotype evolutionary rate apart from the other circulating genotypes being maybe the explanation for the difference between values.

Related to the ancestor, there is only one previous report of the tMRCA for all the existing CTV genotypes [14] in which the ancestor of the clade corresponding to the NC genotype (Group 5 Nolasco’s typing system) was located between 37 and 431 years before 2010 (1579–1973). Despite the broad range obtained by the authors, which may simply reflect the heterogeneity in divergence times for the different CTV genotypes on which the calculations were based, our results are consistent with this.

The continuous movement of infected material, probably in an illegal manner despite the strict sanitary programs of every citrus-growing country, allows this virus genotype to spread continuously. Although this is the first report of the most probable dissemination dates and geographic locations of the CTV-NC genotype, our calculations predict that the virus was present 2–4 years before the first report of this genotype in every country where Group 5 of Nolasco’s typing system was used to report it [42,43]. This result is consistent with a scenario in which the virus remains undetected until the number of infections reaches a detection threshold.

With respect to the spatio-temporal dynamics of the genotype in Uruguay, our results are in agreement with the two different introductions of the virus in the country, and it seems that the population size is growing since the second entrance of the genotype in Uruguay. The local expansion after the second introduction of the virus are confirmed by the high prevalence of this genotype in the country [13]. This population growth could be in agreement with the increment of the number of naïve citrus trees in Uruguayan orchards to encompass the citrus industry increase. Nevertheless, to confirm that the population is still growing, further analyses and continuous vigilance of citrus trees is needed. It has also been shown that temperature has an impact in virus population dynamics, favoring the spreading of thermo-tolerant variants at the expense of a diminished replication of other variants [44]. However, to confirm this hypothesis, further analyses are needed.

## 5. Conclusions

CTV has been moving around the world since the origin of modern citrus-growing techniques. The movement of contaminated material and the local dispersion of the virus by aphids after its arrival to a new place, together with an increasing number of naïve trees are the perfect scenario to a new virus genotype to rise. In the present work, we describe for the first time the dispersion pattern of the recently described NC-genotype around the world as well as the date of origin and its evolutionary rate. In particular, we describe a possible scenario to explain a local growth of its population in Uruguayan citrus orchards, which is in agreement with the emergence of a new variant hypothesis. In order to attain a better understanding of the NC genotype, studies about its biological characterization have to be done. These results are of great importance and must be taken into account to develop a genotype-specific cross-protection program against this highly represented genotype in Uruguay.

## Supplementary Materials

The following are available online at www.mdpi.com/1999-4915/9/10/272/s1.
