# Supplementary material for: The Evolutionary History and Spatiotemporal Dynamics of the NC Lineage of Citrus Tristeza Virus
Source: Viruses. 2017 Oct 12;9(10):272. doi: 10.3390/v9100272 (PMC5691624; doi:10.3390/v9100272)
Supplement: Supplementary file 1 [file viruses-09-00272-s001.zip › TableS1.docx]

|  |  |  |  |  |  |  |  |  |  |
| --- | --- | --- | --- | --- | --- | --- | --- | --- | --- |
|  | Accession Number | Collection Date | Country | Country code (ISO 3166-1) |  | Accession Number | Collection Date | Country | Country code (ISO 3166-1) |
|  | AY660009 | 1995 | Madeira Island | PT-30 |  | KX257290 | 2015 | Uruguay | UY |
|  | DQ660343 | 2004 | Sao Tomé and Principe | ST |  | KX257291 | 2015 | Uruguay | UY |
|  | DQ660350 | 2004 | Angola | AO |  | KX257292 | 2015 | Uruguay | UY |
|  | DQ660353 | 2004 | Angola | AO |  | KX257293 | 2015 | Uruguay | UY |
|  | EU579354 | 2003 | Brazil | BR |  | KX257294 | 2015 | Uruguay | UY |
|  | EU579363 | 2003 | Brazil | BR |  | KX257295 | 2015 | Uruguay | UY |
|  | FJ446484 | 2008 | China | CN |  | KX257296 | 2015 | Uruguay | UY |
|  | GQ475036 | 1998 | Brazil | BR |  | KX257297 | 2015 | Uruguay | UY |
|  | GQ475038 | 1998 | Brazil | BR |  | KX257298 | 2015 | Uruguay | UY |
|  | GQ475039 | 2003 | Brazil | BR |  | KX257299 | 2015 | Uruguay | UY |
|  | GQ475040 | 2003 | Brazil | BR |  | KX257300 | 2015 | Uruguay | UY |
|  | GQ475041 | 2003 | Brazil | BR |  | KX257301 | 2015 | Uruguay | UY |
|  | JQ655290 | 2009 | Portugal | PT |  | KX257302 | 2015 | Uruguay | UY |
|  | JQ655291 | 2009 | Portugal | PT |  | KX257303 | 2015 | Uruguay | UY |
|  | KC202896 | 2009 | Brazil | BR |  | KX257304 | 2015 | Uruguay | UY |
|  | KC202897 | 2009 | Brazil | BR |  | KX257305 | 2015 | Uruguay | UY |
|  | KC590490 | 2010 | India | IN |  | KX257306 | 2015 | Uruguay | UY |
|  | KC841818 | 1980 | United States | US |  | KX257307 | 2015 | Uruguay | UY |
|  | KC841820 | 1990 | Argentina | AR |  | KX257308 | 2015 | Uruguay | UY |
|  | KC841824 | 1979 | United States | US |  | KX257309 | 2015 | Uruguay | UY |
|  | KF144740 | 2010 | China | CN |  | KX257310 | 2015 | Uruguay | UY |
|  | KF144741 | 2010 | China | CN |  | KX257311 | 2015 | Uruguay | UY |
|  | KF144742 | 2010 | China | CN |  | KX257312 | 2015 | Uruguay | UY |
|  | KF144743 | 2010 | China | CN |  | KX257313 | 2015 | Uruguay | UY |
|  | KF144744 | 2010 | China | CN |  | KX257314 | 2015 | Uruguay | UY |
|  | KF196270 | 2012 | Greece | GR |  | KX257315 | 2015 | Uruguay | UY |
|  | KF196272 | 2012 | Greece | GR |  | KX257316 | 2015 | Uruguay | UY |
|  | KF908013 | 2010 | Greece | GR |  | KX257317 | 2015 | Uruguay | UY |
|  | KF962601 | 2010 | Greece | GR |  | KX257318 | 2015 | Uruguay | UY |
|  | KF962602 | 2010 | Greece | GR |  | KX257319 | 2015 | Uruguay | UY |
|  | KF962603 | 2010 | Greece | GR |  | KX257320 | 2015 | Uruguay | UY |
|  | KF962604 | 2010 | Greece | GR |  | KX257321 | 2015 | Uruguay | UY |
|  | KF962605 | 2011 | Greece | GR |  | KX257322 | 2015 | Uruguay | UY |
|  | KF962606 | 2011 | Greece | GR |  | KX257323 | 2015 | Uruguay | UY |
|  | KF962607 | 2011 | Greece | GR |  | KX257324 | 2015 | Uruguay | UY |
|  | KF962608 | 2011 | Greece | GR |  | KX257325 | 2015 | Uruguay | UY |
|  | KF962609 | 2011 | Greece | GR |  | KX257326 | 2015 | Uruguay | UY |
|  | KF962610 | 2012 | Greece | GR |  | KX257327 | 2015 | Uruguay | UY |
|  | KF962611 | 2012 | Greece | GR |  | KX257328 | 2015 | Uruguay | UY |
|  | KF962612 | 2012 | Greece | GR |  | KX257329 | 2015 | Uruguay | UY |
|  | KF962613 | 2012 | Greece | GR |  | KX257330 | 2015 | Uruguay | UY |
|  | KF962614 | 2012 | Greece | GR |  | KX257331 | 2015 | Uruguay | UY |
|  | KF962615 | 2012 | Greece | GR |  | KX257332 | 2015 | Uruguay | UY |
|  | KF962616 | 2012 | Greece | GR |  | KX257333 | 2015 | Uruguay | UY |
|  | KP268418 | 2006 | Uruguay | UY |  | KX257334 | 2015 | Uruguay | UY |
|  | KP268428 | 1997 | Uruguay | UY |  | KX257335 | 2015 | Uruguay | UY |
|  | KP268429 | 1997 | Uruguay | UY |  | KX257336 | 2015 | Uruguay | UY |
|  | KP268430 | 1997 | Uruguay | UY |  | KX257337 | 2015 | Uruguay | UY |
|  | KP268431 | 1997 | Uruguay | UY |  | KX257338 | 2015 | Uruguay | UY |
|  | KP268469 | 1998 | Uruguay | UY |  | KX257339 | 2015 | Uruguay | UY |
|  | KP268470 | 1998 | Uruguay | UY |  | KX257340 | 2015 | Uruguay | UY |
|  | KP268471 | 1998 | Uruguay | UY |  | KX257341 | 2015 | Uruguay | UY |
|  | KP268472 | 1998 | Uruguay | UY |  | KX257342 | 2015 | Uruguay | UY |
|  | KP268474 | 1998 | Uruguay | UY |  | KX257343 | 2015 | Uruguay | UY |
|  | KP268475 | 1998 | Uruguay | UY |  | KX257344 | 2015 | Uruguay | UY |
|  | KP268476 | 1998 | Uruguay | UY |  | KX257345 | 2015 | Uruguay | UY |
|  | KX257284 | 2015 | Uruguay | UY |  | KX257346 | 2015 | Uruguay | UY |
|  | KX257285 | 2015 | Uruguay | UY |  | KX257347 | 2015 | Uruguay | UY |
|  | KX257286 | 2015 | Uruguay | UY |  | KX257348 | 2015 | Uruguay | UY |
|  | KX257287 | 2015 | Uruguay | UY |  | KX257349 | 2015 | Uruguay | UY |
|  | KX257288 | 2015 | Uruguay | UY |  | KX257350 | 2015 | Uruguay | UY |
|  | KX257289 | 2015 | Uruguay | UY |  |  |  |  |  |
