# Supplementary figures and images for: The Evolutionary History and Spatiotemporal Dynamics of the NC Lineage of Citrus Tristeza Virus
Source: Viruses. 2017 Oct 12;9(10):272. doi: 10.3390/v9100272 (PMC5691624; doi:10.3390/v9100272)

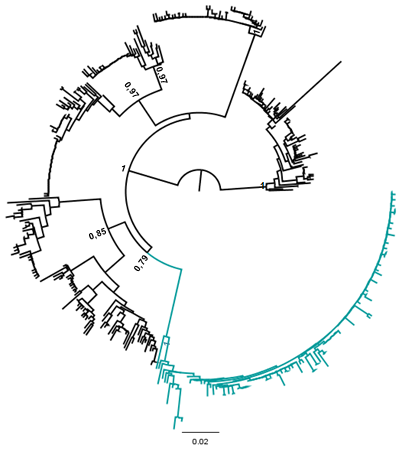

Supplement: Supplementary file 1 [file viruses-09-00272-s001.zip › Figure S1.tif]
